# map3C: a computational tool for processing multiomic single-cell Hi-C data

**DOI:** 10.1093/bioinformatics/btag562

**Published:** 2026-07-29

**Authors:** Joseph Galasso, Ye Wang, Frank Alber, Jason Ernst, Chongyuan Luo

**Affiliations:** Bioinformatics Interdepartmental Program, University of California, Los Angeles, Los Angeles, CA 90095, United States; Department of Biological Chemistry, David Geffen School of Medicine, University of California, Los Angeles, Los Angeles, CA 90095, United States; Department of Human Genetics, University of California, Los Angeles, Los Angeles, CA 90095, United States; Bioinformatics Interdepartmental Program, University of California, Los Angeles, Los Angeles, CA 90095, United States; Institute of Quantitative and Computational Biosciences (QCBio), University of California, Los Angeles, Los Angeles, CA 90095, United States; Department of Pathology, David Geffen School of Medicine, University of California, Los Angeles, Los Angeles, CA 90095, United States; Department of Microbiology, Immunology, and Molecular Genetics, University of California, Los Angeles, Los Angeles, CA 90095, United States; Institute of Quantitative and Computational Biosciences (QCBio), University of California, Los Angeles, Los Angeles, CA 90095, United States; Department of Microbiology, Immunology, and Molecular Genetics, University of California, Los Angeles, Los Angeles, CA 90095, United States; Bioinformatics Interdepartmental Program, University of California, Los Angeles, Los Angeles, CA 90095, United States; Department of Biological Chemistry, David Geffen School of Medicine, University of California, Los Angeles, Los Angeles, CA 90095, United States; Department of Computational Medicine, David Geffen School of Medicine, University of California, Los Angeles, Los Angeles, CA 90095, United States; Computer Science Department, Henry Samueli School of Engineering and Applied Science, University of California, Los Angeles, Los Angeles, CA 90095, United States; Bioinformatics Interdepartmental Program, University of California, Los Angeles, Los Angeles, CA 90095, United States; Department of Human Genetics, University of California, Los Angeles, Los Angeles, CA 90095, United States

## Abstract

**Summary:**

The emergence of multiomic single-cell Hi-C (scHi-C) methods, which simultaneously profile chromatin conformation and other modalities such as gene expression or DNA methylation, creates tremendous opportunities for studying the genome’s structure-function relationships. Existing tools for processing multiomic scHi-C datasets lack certain key functions for downstream bioinformatics analysis. We present map3C, a software tool that incorporates additional key functions. Specifically, we demonstrate that map3C facilitates multiomic scHi-C processing, quality control, and identification of structural variant locations in the genome.

**Availability and implementation:**

map3C is available at https://github.com/luogenomics/map3C and is archived at https://doi.org/10.5281/zenodo.20724719.

## 1 Introduction

Genome-wide chromatin conformation, which is the 3D arrangement of chromatin in the cell nucleus, is profiled with various chromatin conformation capture derived assays such as Hi-C (Lieberman-Aiden *et al.* 2009, Servant *et al.* 2015, Open2C *et al.* 2024). Hi-C uses proximity ligation (PL) to generate DNA fragments containing spatially interacting genomic loci, which are read by high-throughput sequencing (HTS), aligned to a reference genome, and then identified as interactions using software tools such as Pairtools (Open2C *et al.* 2024) and others (Servant *et al.* 2015, Durand *et al.* 2016). These interactions, also called “contacts,” reveal 3D genome features, such as chromatin loops, topologically associating domains, and compartments, which help regulate gene expression (Lieberman-Aiden *et al.* 2009, Dixon *et al.* 2012, Rao *et al.* 2014). In addition to revealing 3D genomic features, the information in Hi-C data has also been shown to be informative for identifying structural variants (SVs) (Wang *et al.* 2020, Song *et al.* 2022, Wang *et al.* 2022).

While originally developed as a bulk assay, recent studies have developed single-cell Hi-C (scHi-C) assays to investigate 3D genomic features at the single cell level (Nagano *et al.* 2013, 2017, Ramani *et al.* 2017). Building upon scHi-C, a number of additional assays have integrated profiling chromatin conformation with other genomic modalities to create “multiomic” assays (Tan *et al.* 2018, Lee *et al.* 2019, Preissl *et al.* 2023, Qu *et al.* 2023, Wu *et al.* 2024, Zhou *et al.* 2024a, Chang et al. 2025). For instance, LiMCA simultaneously profiles transcription and chromatin conformation (Wu *et al.* 2024), while snm3C-seq simultaneously profiles methylation and chromatin conformation (Lee *et al.* 2019). Multiomic scHi-C creates new bioinformatics challenges and opportunities for integrative analysis. While a few tools have been developed for processing multiomic scHi-C data (Tan *et al.* 2018, Lee *et al.* 2019, Liu *et al.* 2021), challenges still remain.

One challenge is that most reported multiomic scHi-C assays, including LiMCA and snm3C-seq (Tan *et al.* 2018, Lee *et al.* 2019, Liu *et al.* 2023, Qu *et al.* 2023, Wu *et al.* 2024), use a variation of the PL approach that introduces alignment artifacts in HTS reads (Wu *et al.* 2019). Similar artifacts, though generated by a different mechanism, are also present in single cell methylation data, which motivated the development of a computational method, scBS-map, to remove them (Wu *et al.* 2019). However, no multiomic scHi-C contact-calling tool currently incorporates this functionality. A second challenge is that none of the previously reported contact-calling tools for snm3C-seq data, TAURUS-MH (Lee *et al.* 2019) and YAP (Liu *et al.* 2021) provide standard sequencing quality control (QC) metrics for filtering out low-quality alignments. A third challenge is that no multiomic scHi-C tool calls SVs at 1 bp genomic resolution. The feasibility of this for bulk Hi-C data was established with the HiNT-TL tool (Wang *et al.* 2020).

To simultaneously address these challenges, we developed map3C. map3C integrates multiomic scHi-C contact-calling via Pairtools algorithms (Open2C *et al.* 2024) with multimapping trimming, QC metric reporting, and reporting of likely SV breakpoints at 1 bp genomic resolution with a variant of HiNT-TL’s algorithm (Wang *et al.* 2020). We present examples demonstrating the utility of these features in the context of multiple LiMCA (Wu *et al.* 2024) and snm3C-seq (Lee *et al.* 2019) datasets from a diverse set of mouse and human tissues.

## 2 Implementation

**Figure 1:**
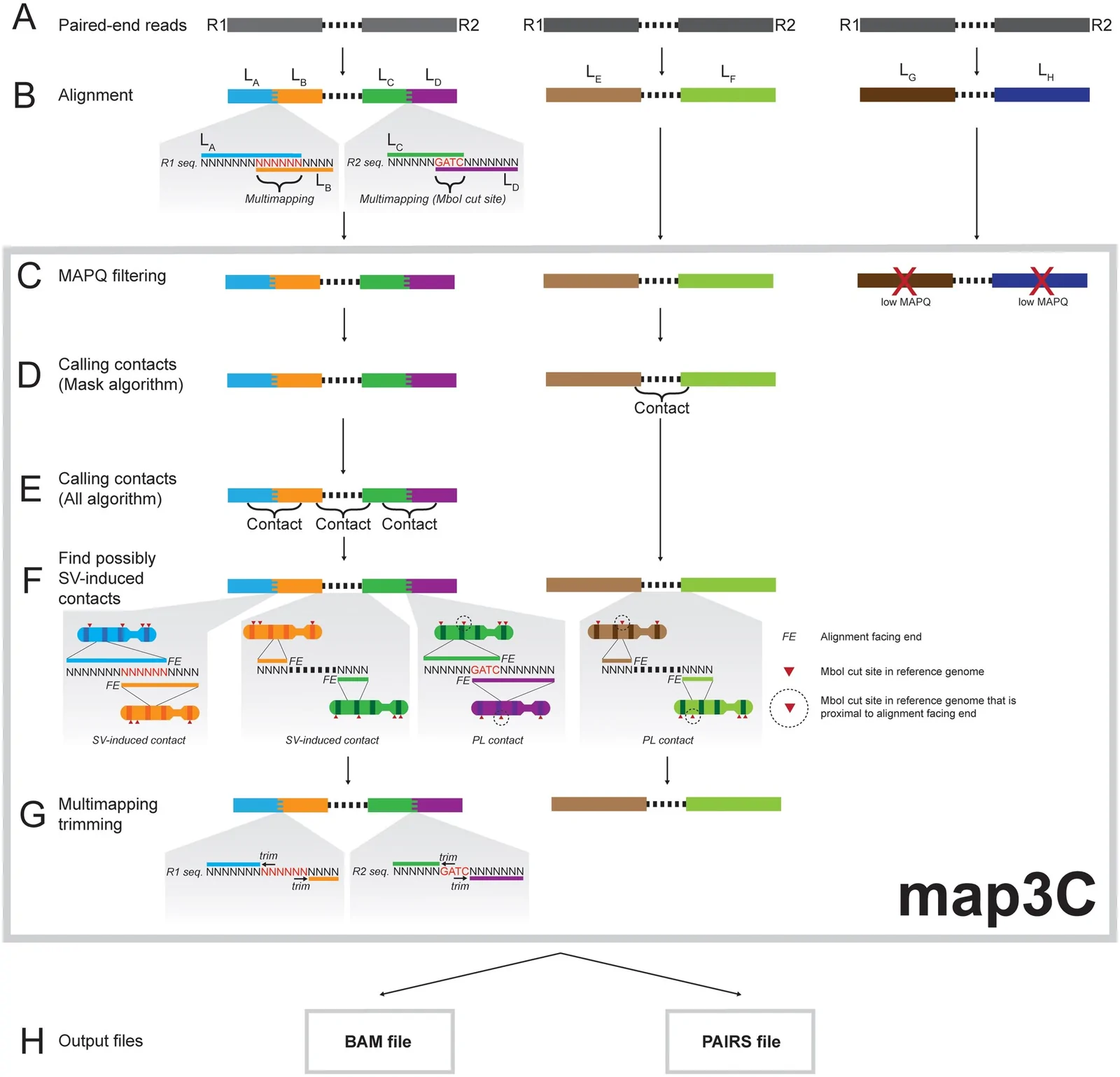
Overview of map3C. (A) Paired-end reads for multiomic scHi-C assays. (B) Reads are aligned to specific loci (L_A_, L_B_, L_C_, L_D_, L_E_, L_F_, L_G_, L_H_) in the reference genome. In this example, loci are all on different chromosomes. L_A_, L_B_, L_C_, and L_D_ alignments are soft-clipped, and the L_A_/L_B_ and L_C_/L_D_ junctions display multimapping. The L_C_/L_D_ junction’s multimapping is induced by an RE cut site. (C) map3C filters out alignments with low MAPQ scores. (D) map3C calls contacts with the Pairtools mask algorithm. (E) map3C optionally calls contacts with the Pairtools all algorithm. (F) map3C analyzes contacts to determine if the alignments’ facing ends are proximal to RE cut sites (represented as red triangles, which are circled if proximal to a facing end). If not, map3C labels these contacts as being more likely induced by SVs. (G) Multimapping portions of adjacent soft-clipped alignments are trimmed. (H) map3C outputs trimmed alignments in a BAM file and contacts in a PAIRS file, with annotations about RE cut site proximity.

map3C takes as input paired end reads locally aligned to a reference genome and produces as output chromatin contacts, which are annotated if they are potentially induced by SVs, and alignments with filtered-out mapping artifacts (Fig. 1). Each paired-end read consists of a forward (R1) and a reverse (R2) read from the input DNA sequences (Fig. 1A). If these reads are not bisulfite-converted, map3C requires them to be aligned with BWA MEM (Li 2013) or BWA MEM2 (Vasimuddin *et al.* 2019), as these are recommended aligners by Pairtools (Fig. 1B). However, if the reads were generated by assays that use bisulfite conversion to profile methylation, such as snm3C-seq (Lee *et al.* 2019), map3C requires that the reads be aligned by Biscuit (Zhou *et al.* 2024b) or BSBolt (Farrell *et al.* 2021), which are designed to handle bisulfite conversion and are based on BWA MEM (Fig. 1B). map3C filters out low-quality alignments based on their MAPQ score (Fig. 1C).

map3C processes alignments through the Pairtools “mask” algorithm (Fig. 1D) and optionally the Pairtools “all” algorithm to generate contacts (Open2C *et al.* 2024) (Fig. 1E). The mask algorithm handles cases where two spatially interacting genomic loci are present in a read pair, while the all algorithm handles cases where more than two spatially interacting genomic loci are present (Open2C *et al.* 2024).

map3C finds and annotates contacts that might be caused by SVs (Fig. 1F). These contacts’ alignments are distal from a restriction enzyme (RE) cut site in the reference genome. SVs cause enrichment of contacts distal from RE cut sites, as some read pairs intersect SV breakpoints. Furthermore, if an SV-induced contact’s alignments are adjacent and “soft-clipped,” the junction between the two alignments can reveal the SV breakpoint at 1 bp resolution (Rausch *et al.* 2012, Chen *et al.* 2016, Wang *et al.* 2020). Soft-clipped alignments are alignments to only a portion of a read, which are produced by aligners for which map3C is compatible. This approach for identifying contacts that are more likely to be caused by SVs is similar to that of HiNT-TL but has not been previously integrated into multiomic scHi-C software. Furthermore, map3C reports all contacts that are more likely to be caused by SVs, in contrast to HiNT-TL, which only reports interchromosomal SVs (Wang *et al.* 2020).

map3C removes alignment artifacts at ligation junctions between spatially interacting genomic loci (Fig. 1G). Some of these artifacts are caused by sticky-end PL, which is used in several multiomic scHi-C assays since it has efficiency advantages over the alternative blunt-end PL (Lieberman-Aiden *et al.* 2009, Nagano *et al.* 2013, 2017, Rao *et al.* 2014). Sticky-end ligation creates a single RE motif at the junction between two ligated loci. Since each locus borders a motif in the reference genome, aligners may multimap the motif to each locus (Fig. 1A), but the motif can only derive from one locus *in vivo*. map3C removes all multimapping between adjacent soft-clipped alignments from the same read (Fig. 1G) to eliminate inaccurate alignments.

map3C produces two main output files (Fig. 1H). The first is a BAM file containing multimapping-trimmed alignments that are above a minimum MAPQ threshold, which can be customized by the user. The second is a PAIRS file containing contacts (Open2C *et al.* 2024). The contacts in the PAIRS file are annotated if they fit criteria for being more likely to be induced by SVs.

## 3 Example cases

### 3.1 Case 1: finding SV breakpoints in LiMCA data with map3C

We applied map3C to identify SV breakpoints in K562, a myelogenous leukemia cell line. Specifically, we used map3C to call contacts on 63 K562 cells profiled by LiMCA (Wu *et al.* 2024) to identify 358,716 possible bp-resolution SV breakpoints. We note that many of these called breakpoints may not actually be due to SVs and are instead due to technical or alignment artifacts commonly associated with HTS and Hi-C (Imakaev *et al.* 2012, Dixon *et al.* 2018, Koboldt 2020). Thus, we applied a similar approach as HiNT-TL to identify higher-confidence 1 bp breakpoints by intersecting the 1 bp breakpoints with low resolution SVs called from the merged LiMCA contact matrices of all K562 cells (Wang *et al.* 2020). map3C has additional dataset compatibility and functional advantages in this analysis over HiNT-TL. HiNT-TL is not compatible with sticky-end PL Hi-C datasets, such as LiMCA. Furthermore, HiNT-TL only identifies bp-resolution breakpoints for interchromosomal SVs, whereas map3C identifies bp-resolution breakpoints for both intrachromosomal and interchromosomal SVs. We applied EagleC (Wang *et al*. 2022), a method for calling SV breakpoints at 5 kb resolution from Hi-C contact frequencies, to the entire set of K562 contacts, generating 112 breakpoints. The EagleC breakpoints were intersected with the map3C breakpoints to generate a final list of 61 1 bp breakpoints, which were within the boundaries of 44 unique EagleC-identified SVs (Fig. S1, available as supplementary data at *Bioinformatics* online). Of these 61 bp-resolution breakpoints, 23 were validated by intersection within a  ± 10 bp window of 4,273 SV calls from whole genome sequencing (WGS) of K562 (Dixon *et al.* 2018). Assuming that the WGS breakpoints are ground truth, we computed a precision of 0.30 and a recall of 0.0033 after deduplicating the map3C breakpoints to remove highly proximal breakpoints that may reflect technical or alignment artifacts rather than distinct rearrangements. The low recall is expected due to the intersection of map3C breakpoints with EagleC-identified SVs, which contained orders of magnitude fewer SVs than WGS. The true precision could be much higher due to clonal evolution of K562 creating unique SVs that were not present in the WGS dataset. Clonal evolution has been reported for other cell lines (Ben-David *et al.* 2018, Petljak *et al.* 2019).

Since map3C effectively employs WGS-style 1 bp breakpoint identification in an optimized manner for multiomic scHi-C, filtering techniques developed for WGS to remove low-confidence breakpoints from WGS could be applied to map3C breakpoints. For instance, a hallmark of breakpoints resulting from sequencing or analysis artifacts is their co-occurrence in multiple WGS datasets for different cell lines (Dixon *et al.* 2018, Koboldt 2020). Thus, we determined if the EagleC 5 kb-resolution K562 breakpoints also had evidence of being breakpoints in GM12878, a non-cancer cell line, by applying map3C to LiMCA data for 221 GM12878 cells (Wu *et al.* 2024) (Fig. S1, available as supplementary data at *Bioinformatics* online). Of the K562 breakpoints identified with EagleC, 93% have no supporting GM12878 contacts despite the GM12878 dataset having 3.1× more HTS reads than the K562 dataset, thus suggesting most breakpoints are not driven by technical artifacts that are common across cell lines. Ideally, this filtering technique would be done with a larger set of tissues or cell lines.

### 3.2 Case 2: improving alignment accuracy with map3C multimapping trimming

We next demonstrate that map3C multimapping trimming can remove inaccurate alignments. In a LiMCA-profiled mouse olfactory epithelium cell (Wu *et al.* 2024), 26% of 3,342,056 total soft-clipped alignments had multimapping, and map3C trimmed out 8% (std = 4%) of their spans along their respective reads on average. Similarly, in a snm3C-seq profiled mouse embryonic stem cell (mESC) (Lee *et al.* 2019), 30% of 251,356 total soft-clipped alignments had multimapping and map3C trimmed out 11% (std = 7%) of their read spans on average. To provide evidence that multimapping contributes to inaccurate alignments, we identified LiMCA alignments that intersected ≥3 SNPs and had conflicting haplotype assignments between these SNPs (Supplementary Methods). A potential explanation for the conflicting SNP haplotype assignments in such cases is that the SNP observations from multimapping regions are inaccurate. Consistent with this explanation, in 40 LiMCA-profiled DBA/2J × C57BL/5J cells and 40 LiMCA-profiled CAST/EiJ × C57BL/6J cells (Wu *et al.* 2024), we found 5,810 and 34,471 alignments, respectively, that fit the above criteria before map3C multimapping trimming. After trimming, 77% of DBA/2J × C57BL/5J and 79% of CAST/EiJ × C57BL/6J conflicts were resolved, as remaining SNPs agreed on a haplotype (Fig. S2A and S2B, available as supplementary data at *Bioinformatics* online). For comparison, for each of these alignments, if we assign haplotypes after randomly excluding the same number of SNP observations that were removed by multimapping trimming, the conflict reduction rate drops to 28% on average (std = 0.6%) for DBA/2J × C57BL/5J (Fig. S2A, available as supplementary data at *Bioinformatics* online) and 29% on average (std = 0.2%) for CAST/EiJ × C57BL/6J (Fig. S2B, available as supplementary data at *Bioinformatics* online).

### 3.3 Case 3: map3C QC of snm3C-seq data to enable single-cell IGM

We show that map3C improves computational 3D chromatin modeling by removing spurious alignments from bisulfite-converted multiomic scHi-C data via MAPQ filtering. MAPQ is not reported in other pipelines for this data type despite it being a standard QC metric that is output by aligners like BWA MEM (Li 2013). To assess contact quality in snm3C-seq, we analyzed the intrachromosomal-to-interchromosomal contact ratio in 351 mESCs. The same input reads for each cell were processed with map3C, TAURUS-MH, and YAP. map3C yielded a higher ratio (mean = 3.16 ± 1.23) than for TAURUS-MH (mean = 0.98 ± 0.26) or YAP (mean = 1.10 ± 0.31) on the same dataset (Fig. S3A, available as supplementary data at *Bioinformatics* online), closer to the 4.22 ratio reported by the 4DNucleome pipeline (Reiff *et al.* 2022). The interchromosomal contacts reported by map3C are significantly lower than TAURUS-MH and YAP (*P *< .001, one-sided Mann-Whitney U) in Fig. S3A, available as supplementary data at *Bioinformatics* online. Improvements from map3C processing are evident in 3D genome structures generated with our modified integrative genome modeling (IGM) software (Boninsegna *et al.* 2022). Constructing 3D models that satisfy all chromatin contacts indicates self-consistent, high-quality snm3C-seq data, whereas physically incompatible spurious contacts that cannot be simultaneously realized in 3D increase the fraction of violated constraints per model (restraint-violation rate) and signal poor data quality. Models from map3C-derived contacts showed the lowest restraint-violation rate (Median: 0.004, Mean: 0.006 ± SD: 0.01), outperforming TAURUS-MH (Median: 0.051, Mean: 0.057 ± SD: 0.041) and YAP (Median: 0.037, Mean: 0.039 ± SD: 0.031) (Fig. S3B, available as supplementary data at *Bioinformatics* online). The restraint-violation rate reported by map3C is significantly lower than TAURUS-MH and YAP (*P *< .001, two-sided Mann-Whitney U) in Fig. S3B, available as supplementary data at *Bioinformatics* online. TAURUS-MH and YAP-derived IGM structures appeared to have higher chromatin density compared to map3C-derived structures (Fig. S3C, available as supplementary data at *Bioinformatics* online). To better quantify this, we calculated the cumulative chromatin density across five concentric volumetric shells from the nuclear center to the periphery (Fig. S3D, available as supplementary data at *Bioinformatics* online). Models generated from TAURUS-MH and YAP contacts showed significantly higher chromatin density in the inner shells (*P *< .001, two-sided Mann-Whitney U). In YAP and TAURUS-MH-derived models, nearly 83% and 88% of all chromatin was located within the two innermost shells, with little chromatin occupying the two outermost shells (Fig. S3D, available as supplementary data at *Bioinformatics* online). Similarly, the innermost shell of TAURUS-MH and YAP-derived models showed 75% and 55% higher chromatin density (*P *< .001, two-sided Mann-Whitney U) than map3C-derived models, respectively (Fig. S3E, available as supplementary data at *Bioinformatics* online). The more evenly distributed chromatin density in map3C-derived models indicates that map3C structures occupy the full nuclear volume more realistically (Fig. S3D and S3E, available as supplementary data at *Bioinformatics* online).

We benchmarked structural accuracy against DNA seqFISH+ (Takei *et al.* 2025) imaging data in mESC by comparing (i) the average nuclear radial positioning of genomic regions and (ii) interchromosomal contact probability (ICP), a feature linked to transcriptional activity (Boninsegna *et al.* 2022, Yildirim *et al.* 2022, 2023). Models from map3C contacts more closely recapitulated the observed radial profiles (Fig. S3F, available as supplementary data at *Bioinformatics* online) and showed substantially better agreement with imaging-derived ICP values compared to YAP and TAURUS-MH (Fig. S3G, available as supplementary data at *Bioinformatics* online; Spearman *r*: map3C = 0.76, YAP = 0.45, TAURUS-MH = 0.51), demonstrating that map3C’s stringent alignment filtering enhances single-cell model fidelity.

## Supplementary Material

btag562_Supplementary_Data

## Data Availability

The map3C software is available from https://github.com/luogenomics/map3C and the version used in this article is archived at https://doi.org/10.5281/zenodo.20724719. The LiMCA dataset analyzed in this study is available from the NCBI Gene Expression Omnibus (GEO) under accession GSE240128, the snmC-seq2 dataset under accession GSE245367, and the snm3C-seq dataset under accession GSE124391. The LiMCA phased SNP databases were obtained from GitHub (https://github.com/zhang-jiankun/LiMCA/raw/refs/heads/main/data/mouse_snps/dba2.txt.gz and https://github.com/zhang-jiankun/LiMCA/raw/refs/heads/main/data/mouse_snps/cast.txt.gz). The lambda phage genome was downloaded from GenBank (https://www.ncbi.nlm.nih.gov/nuccore/J02459.1?report=fasta) and the mm10 excluded regions were downloaded from GitHub (https://github.com/Boyle-Lab/Blacklist/raw/refs/heads/master/lists/mm10-blacklist.v2.bed.gz).
